# Antibody Response Following COVID-19 Vaccination in Malaysian Cancer Patients and Healthy Individuals

**DOI:** 10.7759/cureus.73528

**Published:** 2024-11-12

**Authors:** Chin Vern Song, Ros Suzanna Ahmad Bustamam, Gan Gin Gin, Marniza Saad, Nur Fadhlina Abdul Satar, Alagu Manthiram Ramasamy, I-Ching Sam, Yek-Ching Kong, Harenthri Devy Alagir Rajah, Yoke Fun Chan, Jolene Yin Ling Fu, Cheng Siang Tan, Mahmoud Danaee, Cheng Har Yip, Carla H Van Gils, Nirmala Bhoo-Pathy

**Affiliations:** 1 Julius Center, UMC (University Medical Center) Utrecht, Utrecht, NLD; 2 Department of Radiotherapy and Oncology, Hospital Kuala Lumpur, Kuala Lumpur, MYS; 3 Faculty of Medicine, Universiti Malaya, Kuala Lumpur, MYS; 4 Faculty of Medicine and Health Sciences, Universiti Malaysia Sarawak, Kota Samarahan, MYS; 5 Department of Breast Cancer Surgery, Sime Darby Medical Center, Subang Jaya, MYS

**Keywords:** antibody, cancer, cohort study, covid-19, vaccine

## Abstract

Introduction: There is a lack of real-world evidence on direct comparisons between COVID-19 vaccines in multiethnic low- and middle-income settings. Cancer patients have an impaired vaccine response due to the disease itself or the effects of treatment. Hence, identifying the best vaccine to use for cancer patients is important. We aimed to compare the antibody response between cancer patients and healthy individuals following COVID-19 vaccination and assess seroconversion rates, vaccine efficacy, and the impact of sex on antibody response, as well as document adverse events in cancer patients.

Materials and methods: A prospective cohort study of cancer patients and healthy individuals receiving vaccines was conducted in Malaysia. All participants were aged 18 or above at recruitment and received at least two doses of vaccine. We excluded patients who had missing serum antibody data post-first dose and post-second dose. Sociodemographic and clinical data were collected at baseline, prior to vaccination. Data on self-reported breakthrough infection was collected at six months. Multivariable linear mixed-effects regression models were used to investigate the association between the type of vaccine and serum IgG titer.

Results: A total of 389 patients with solid (n=276, 71.0%) or hematologic cancers (n=113, 29.0%) were included, along with 246 healthy individuals. Most cancer patients received BNT162b2 (n=358, 92.0%), followed by AZ1222 (n=19, 4.9%) and Coronavac (n=12, 3.1%). Most healthy individuals received BNT162b2 (n=151, 61.4%), followed by Coronavac (n=95, 38.6%). Vaccination, after adjustment for confounders (pre-vaccine infection, age, ethnicity, comorbidity, timepoint, income, cancer type, and booster), with Coronavac was associated with lower log IgG titer (-3.09 U/ml, 95% confidence interval=-4.37 to -1.80, p<0.01) than that of BNT162b2 in patients with cancer and also lower log IgG titer (-2.64 U/ml, 95% confidence interval=-2.97 to -2.30, p<0.01) than that of BNT162b2 in healthy individuals. No effect modification by sex was observed. Among the cancer cohort, 76 patients (19.5%) reported breakthrough infections after vaccination, while 33 (13.4%) participants in the healthy cohort reported breakthrough infections after vaccination. Coronavac was associated with greater odds of breakthrough infection among healthy individuals (odds ratio=7.34 compared to BNT162b2, confidence interval=1.40 to 33.49, p=0.02).

Conclusion: Vaccination with BNT162b2 yields higher IgG titer than Coronavac in all groups and fewer breakthrough infections in healthy subjects. The effect of vaccination is not modified by sex.

## Introduction

Vaccines are an important part of the global response to the COVID-19 pandemic, as they prevent and reduce the severity of infection. Many different types of COVID-19 vaccines have been produced by different manufacturers, which have different mechanisms of action [1]. Prior evidence has shown that these vaccines are safe and effective in the general population [2].

Cancer patients are particularly vulnerable to COVID-19 infection. They have a greater risk of mortality following COVID-19 compared to the general population due to a weakened immune system attributed to both the disease and its treatments, e.g. chemotherapy [3]. Furthermore, the effectiveness of COVID-19 vaccines in cancer patients may be reduced because of anti-cancer medications [4]. A prior meta-analysis investigating the effectiveness of COVID-19 vaccines in individuals with cancer observed lower seroconversion in patients with solid cancer compared to healthy controls (pooled relative risk: 0.90, 0.88-0.93), and even more so in patients with hematological cancer (pooled relative risk: 0.63, 0.57-0.69) [5]. The relative vulnerability of cancer patients to COVID-19, along with poorer response to vaccination, implies that research on optimizing vaccination strategies in cancer patients is essential.

Previous studies on COVID-19 vaccination were conducted in high-income countries with limited vaccine diversity, which makes it difficult to directly compare different types of vaccines [1,5]. Furthermore, at the global level, there has been very little planning for systematic collection of data from patients with cancer receiving COVID-19 vaccines, particularly in low- and middle-income countries (LMICs) [6]. As a result, real-world evidence on the effectiveness of COVID-19 vaccines is scarce in LMICs as most of the prior knowledge has been derived from clinical trials [7]. Socioeconomic factors, which may significantly impact vaccine effectiveness [8], and the understudied effect of sex on vaccine response are critical areas that require more research. Addressing these factors is essential to achieving equitable healthcare outcomes [9]. To address these knowledge gaps, we investigated the association of vaccine types (BNT162b2, AZ1222, Coronavac) with antibody response in a multi-ethnic population from a middle-income setting (Malaysia), including both healthy individuals and patients with cancer. The primary objective of our study was to compare the antibody response between cancer patients and healthy individuals following COVID-19 vaccination. We also had the secondary objectives of assessing seroconversion rates, vaccine efficacy, and the impact of sex on antibody response, as well as documenting adverse events in cancer patients.

## Materials and methods

We analyzed data from two prospective cohort studies on individuals with and without cancer, the Immune Response Following COVID-19 Vaccination in Malaysians With Cancer (IRESPOND) cohort, and the ASEAN Sero-Surveillance Study on COVID-19 vaccines (ASSeSS) cohort. In the cancer (IRESPOND) cohort, participants were recruited from the vaccine centers of two tertiary hospitals in Kuala Lumpur, Malaysia, namely the Universiti Malaya Medical Center (UMMC) and Hospital Kuala Lumpur, which provided vaccination to their catchment populations. The study received ethics approval from the Medical Ethics Committee of Universiti Malaya Medical Centre. The approval number of the ASSeSS cohort was 2021728-10423. The approval number of the IRESPOND cohort was NMRR-21-978-60010.

**Figure 1:**
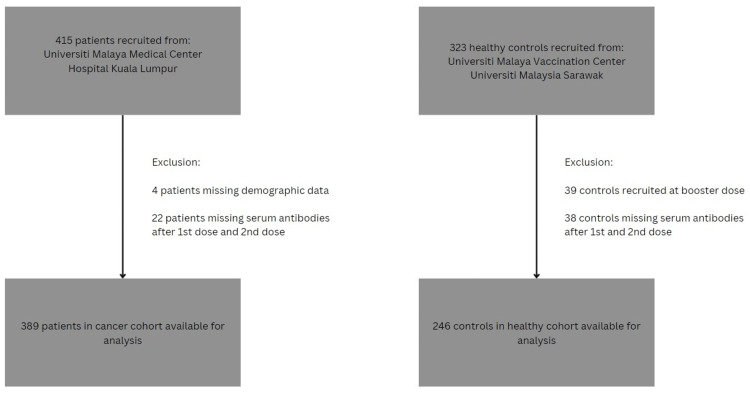
Flow chart of patient and control selection

All participants were aged 18 or above at recruitment and received at least two doses of vaccine (fully vaccinated). Between May 2021 and October 2021, adults receiving cancer care, encompassing both newly diagnosed patients and those on follow-up, were included. The cancer cohort included patients of all cancer types and all cancer stages. The healthy cohort was established independently between March 2021 and March 2022 and comprised adults without cancer. They included volunteers who were vaccinated at the UMMC vaccine center and healthcare workers from the University Malaysia Sarawak. The timing of recruitment coincided with the fourth COVID-19 wave to hit Malaysia (June 2021 to January 2022) [10]. We excluded participants who did not have blood sample data for both the first dose and the second dose. The process of participant selection is described in Figure 1. The study received ethical approval from the relevant institutional ethical review boards.

The National COVID-19 Immunisation Programme in Malaysia used three different vaccines, namely the BNT162b2 (Pfizer), AZ1222 (AstraZeneca), and Coronavac (Sinovac) vaccines, which were provided for free, encompassing two doses plus a booster. The second dose of the vaccine was administered three weeks after the first dose for BNT162b2 and Coronavac, while the second dose was given 12 weeks after the first dose for AZ1222. Some patients received a booster after the second dose.

We collected participants’ baseline data through face-to-face interviews at recruitment. In patients with cancer, we collected data including age in years (continuous), sex (male/ female), ethnicity (Malay/ Chinese/ Indian/ Other), income (low, middle, high [11]), vaccine type (BNT162b2/ AZ1222/ Coronavac), cancer type (solid/ hematologic), and comorbidities (yes/ no). In the healthy individuals, only data on age, sex, ethnicity, vaccine type, and comorbidities were collected. Participants’ blood samples were collected at several time points (TP): pre-first dose (TP1), pre-second dose (TP2), two weeks post-second dose (TP3), six months after first dose TP1 (TP4), and one year after first dose (TP5). Whole-blood samples (10 ml per sampling) were taken by trained medical staff and stored in heparinized vacutainers. Booster status (yes/no) and breakthrough infections (yes/no) were obtained at six months. Sampling timepoint was treated as categorical data (TP1/ TP2/ TP3/ TP4/ TP5).

Antibody response was determined by measuring the participant’s COVID-19 IgG antibody levels using the Elecsys Anti-SARS-CoV-2 S (Roche Diagnostics GmbH, Mannheim, Germany), a quantitative electrochemiluminescence immunoassay. The assay’s sensitivity and specificity have previously been validated [12]. The cutoff for IgG detection (positive seroconversion) was 0.8 U/ml, as stated in the manufacturer’s instructions [13]. Participants were considered to have pre-vaccine COVID-19 infection if they had detectable antibodies before the first dose. The assay’s range of measurements without dilution is 0.4 U/ml to 250 U/ml. Samples were diluted 1/100 to give a maximum measurement of 25000 U/ml in the cancer cohort. Samples were diluted 1/50 to give a maximum measurement of 12500 U/ml in the healthy cohort [14]. As a result, a direct comparison between healthy and cancer cohorts used seroconversion as a binary outcome (yes/no), instead of IgG levels.

We collected data on any possible vaccine-related complications that occurred within eight days of vaccination, after the first and second doses. The data were collected by telephone calls. These include erythema, pain or swelling at the injection site, lymphadenopathy, flu-like symptoms, headaches, chills, fatigue, arthralgia, nausea or vomiting, fever, and diarrhea. Adverse events were graded according to the following scale: grade 1 (mild; does not interfere with activity); grade 2 (moderate; interferes with activity); grade 3 (severe; prevents daily activity); and grade 4 (potentially life-threatening; emergency department visit or admission to hospital). Only moderate adverse events (grade 2 and above) were counted for analysis. At TP4, participants were asked if they were diagnosed or admitted for COVID-19 breakthrough infection after vaccination (TP2 to TP4), which were either confirmed by rapid antigen testing or reverse transcription-polymerase chain reaction for the cancer cohort. This information was, however, not verified against medical records. In the healthy cohort, participants were asked if they had COVID-19 infection after vaccination, the data of which were also not verified against medical records.

Data analysis was conducted using IBM SPSS Statistics for Windows Version 29.0 (IBM Corp., Armonk, NY) and R version 4.2.0 in the R Studio environment (R Foundation for Statistical Computing, Vienna, Austria) [15,16]. Multiple imputation was used to account for missing data, with sociodemographic and medical variables used as predictors. Predictive mean matching was used to impute missing data. Five imputed datasets were created and the results from each dataset were pooled using Rubin’s Rule to obtain the final estimates [17].

Differences between categorical variables were compared using the chi-square test and differences between continuous variables were compared using the Kruskall-Wallis test. Means were reported along with standard deviation (SD), and medians were reported along with percentiles. Our data was multilevel, with patient ID as the cluster variable and the recorded serum IgG titer at each timepoint nested by patient ID. Four serum IgG titer timepoints (TP1, TP2, TP3, and TP4) were included for each patient. Logistic mixed-effects regression was used to investigate the association between the type of vaccine and seroconversion (yes/no). Linear mixed-effects regression modeling was used to investigate the association between the type of vaccine and log-transformed serum IgG titer (continuous outcome) within each cohort. Random-effect variables were participant IDs, whereas all other variables were treated as fixed-effect variables. The models were adjusted for sociodemographic and medical variables including age, ethnicity, comorbidity, sampling timepoint, income, cancer type, booster status, and pre-vaccine infection. Since the cancer cohort included long-term survivors who may have different effects of vaccination compared to recently diagnosed patients, a sensitivity analysis was performed excluding cancer patients who were diagnosed before 2020 and 2021. To test for effect modification we compared models, one with a multiplicative term and one without, using the D1 statistic, which is the multivariate Wald test [18].

Finally, logistic regression analysis was used to investigate the association of vaccine type and serum IgG with breakthrough infection (dichotomous outcome: yes, no). The models were adjusted for sociodemographic and clinical factors including age, ethnicity, comorbidity, sampling timepoint, income, cancer type, and booster status. We excluded patients with pre-vaccine infection, determined by serology, from this analysis.

## Results

A total of 389 cancer patients and 246 healthy individuals were included (Table 1). The median age of the participants in the healthy cohort was lower than the cancer cohort (41 (25th percentile: 34, 75th percentile: 49) vs. 53 (25th percentile: 42, 75th percentile: 62), respectively, p<0.01). The healthy cohort also had a lower proportion of individuals with comorbidities than the cancer cohort (7.3% vs. 36.8%, respectively, p<0.001). The cancer cohort mostly received BNT162b2 (n=358, 92%), followed by AZ1222 (n=19, 4.9%) and Coronavac (n=12, 3.1%). The healthy cohort mostly received BNT162b2 (n=151, 61.4%), followed by Coronavac (n=95, 38.6%). No one in the healthy cohort received AZ1222. In the cancer cohort, 42 patients who received BNT162b2 (11.7%), 15 patients who received AZ1222 (78.9%), and one patient (8.3%) who received Coronavac had a pre-vaccine infection. In the healthy cohort, 10 individuals (6.6%) who received BNT162b2 and two individuals (2.1%) who received Coronavac had a pre-vaccine infection.

**Table 1:** Demographic characteristics of study population

Variables	Cancer patients, n=389	Healthy individuals, n=246
Age, years; median (interquartile range)	53 (42-62)	41 (34-49)
Ethnicity		
Malay	160 (41.1)	77 (31.3)
Chinese	180 (46.3)	108 (43.9)
Indian	38 (9.8)	11 (4.5)
Other	11 (2.8)	50 (20.3)
Sex		
Male	138 (35.5)	103 (41.9)
Female	251 (64.5)	143 (58.1)
Income		
Lowest 40% ( < RM4850)	258 (66.3)	
Middle 40% (RM4850-RM10959)	80 (20.6)	
Highest 20% (>RM10959)	43 (11.1)	
Missing	8 (2.0)	
Vaccine		
BNT162b2	358 (92.0)	151 (61.4)
AZ1222	19 (4.9)	0
Coronavac	12 (3.1)	95 (38.6)
Booster		
Yes	94 (24.2)	98 (39.8)
No	162 (41.6)	148 (60.2)
Missing	133 (34.2)	0
Pre-vaccine infection		
Yes	60 (15.4)	12 (4.9)
No	268 (68.9)	234 (95.1)
Missing	61 (15.7)	0
Breakthrough infection		
Yes	76 (19.5)	33 (13.4)
No	122 (31.4)	213 (86.6)
Missing	191 (49.1)	0
Comorbidity		
Yes	143 (36.8)	18 (7.3)
No	246 (63.2)	228 (92.7)
Cancer type		
Solid	276 (71.0)	
Hematologic	113 (29.0)	
Year of cancer diagnosis		
2021	51 (13.1)	
2020	93 (23.9)	
2019	54 (13.9)	
2018	34 (8.7)	
2017 and before	93 (23.9)	
Missing	64 (16.5)	

Data on antibody response at six months (TP4) was available for 66.3% of all participants in the cancer cohort and 55.3% of all participants in the healthy cohort. Meanwhile, data on antibody response at one year (TP5) was available only in 199 out of 389 patients with cancer (51.2%) and 89 out of 246 healthy individuals (36.2%). In these participants, seroconversion was maintained for everyone at one year. Due to high attrition at TP5, we decided to limit our main analysis to TP4, for which data was available for 66.3% in the cancer cohort and 55.3% in the healthy cohort. At TP5, the mean log serum IgG was 8.05 (standard deviation 1.19) among healthy individuals and 8.068 (standard deviation 2.06) among patients with cancer; p=0.94.

Seroconversion among the cancer cohort and healthy cohort are shown in Table 2. The vast majority (95.1% in the cancer cohort and 100% in the healthy cohort) of individuals exhibited seroconversion after the second dose (TP3). This trend persisted six months later. Given the very low number of participants who did not seroconvert, we were unable to perform further regression analysis on vaccine type and seroconversion as a dichotomous outcome.

**Table 2:** Seroprevalence by type of vaccine among healthy individuals and cancer patients ^1^TP1: pre-first dose, TP2: pre-second dose, TP3: 2 weeks post-second dose, TP4: 6 months post-first dose. ^2^Antibodies considered present if IgG titer was above 0.8 U/ml.

Time point (TP)^1^	BNT162B2		AZ1222		Coronavac	
	Seroprevalence %	N with detectable antibodies^2^/Total with data	Seroprevalence %	N with detectable antibodies^2^/Total with data	Seroprevalence %	N with detectable antibodies^2^/Total with data
Cancer cohort						
TP1	15.0	46/307	86.7	13/15	16.7	1/6
TP2	76.3	273/358	100	19/19	16.7	2/12
TP3	95.8	343/358	100	19/19	66.7	8/12
TP4	95.7	223/233	100	18/18	85.7	6/7
Healthy cohort						
TP1	6.6	10/151			2.1	2/95
TP2	100	151/151			100	95/95
TP3	100	127/127			100	93/93
TP4	100	52/52			100	83/84

**Figure 2:**
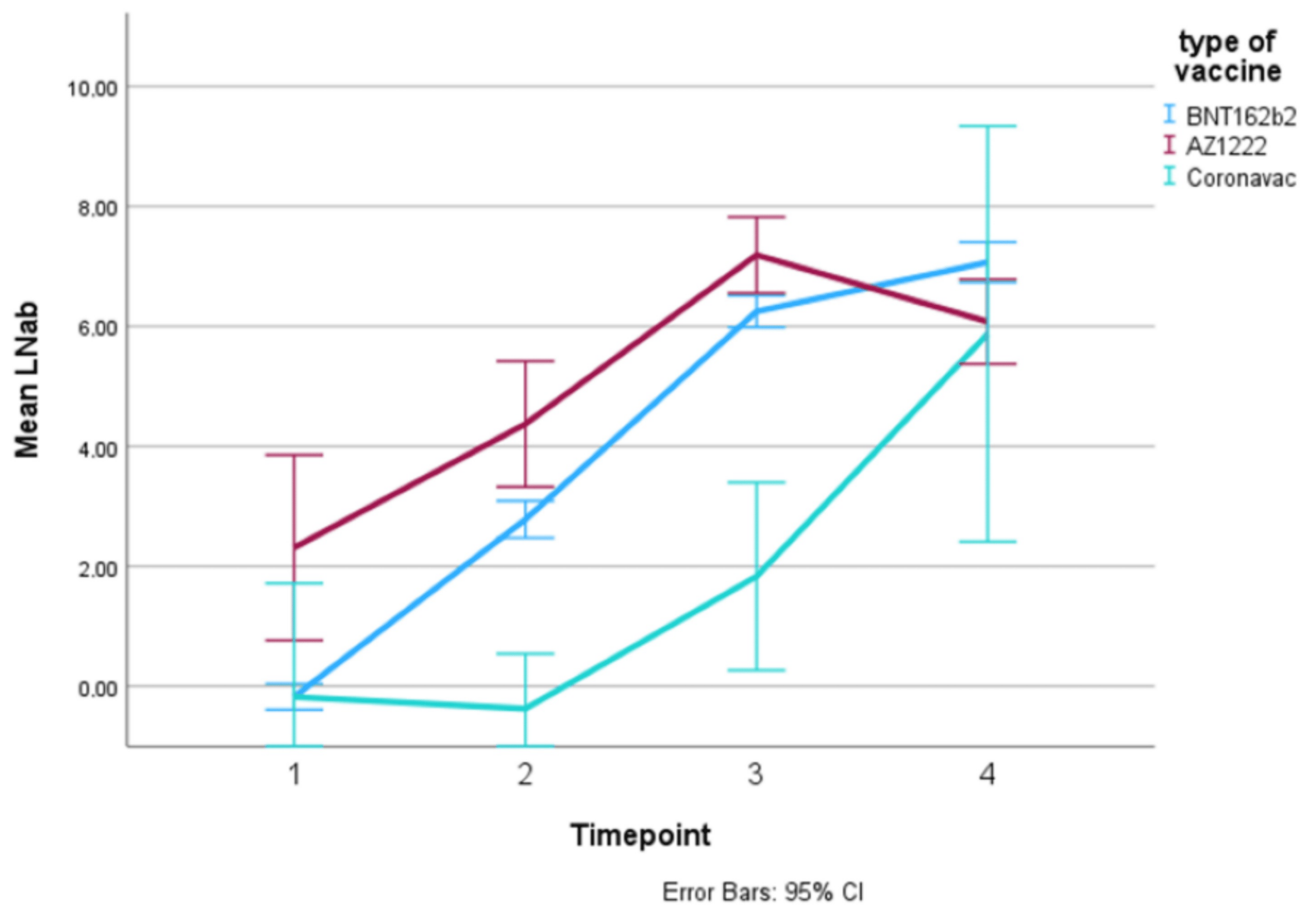
Trend in mean log IgG titer, in antibody units, over time points in the cancer cohort

**Figure 3:**
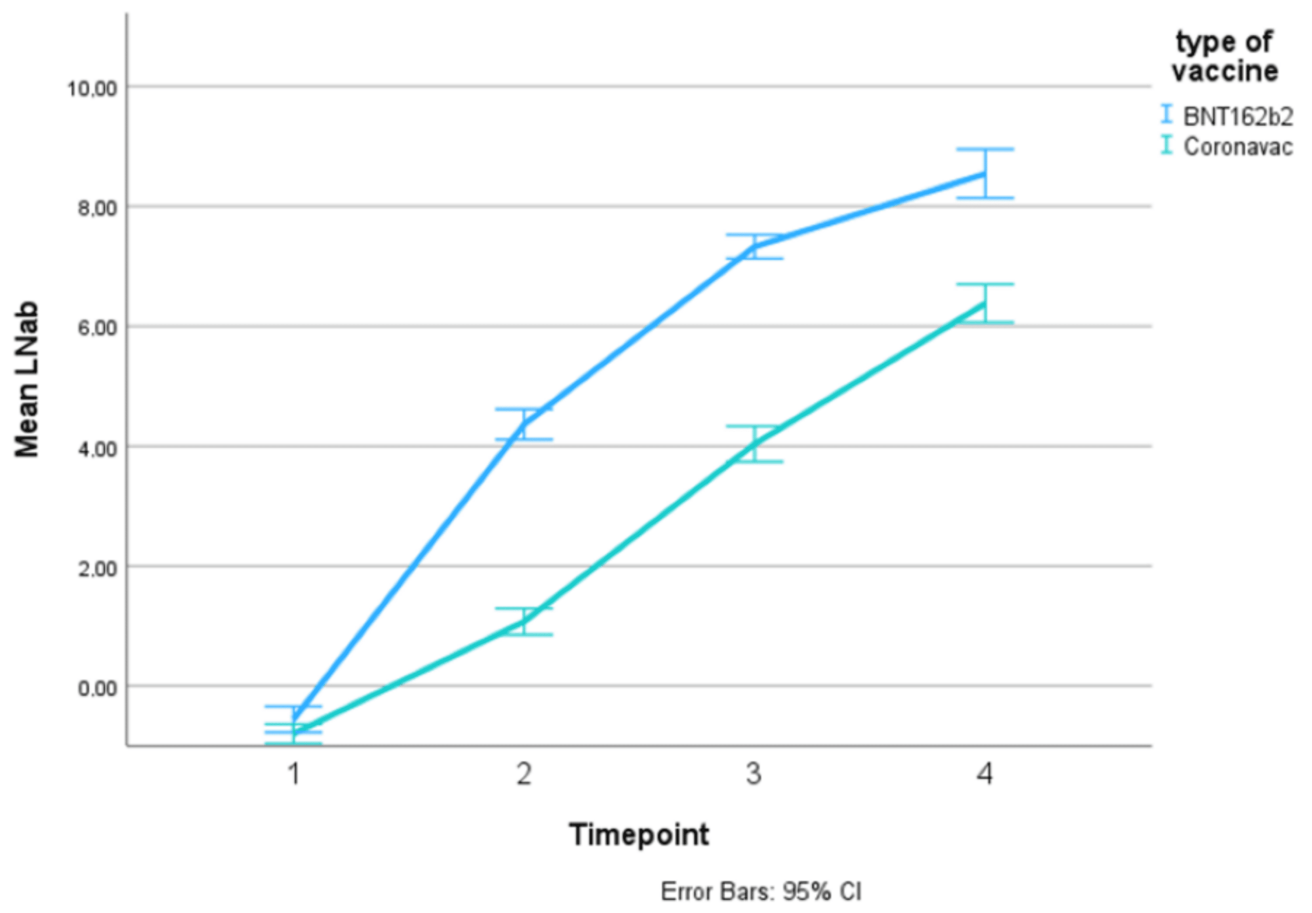
Trend in mean log IgG titer, in antibody units, over time points in the healthy cohort

The overall trend in log serum IgG titer increased over time in both cohorts and in all vaccine types (Figures 2, 3, Table 3). It was noted that cancer patients who were vaccinated with AZ1222 had high mean serum IgG titer at pre-vaccination (TP1). This could be because the majority of AZ1222 patients had pre-vaccine infection. Patients vaccinated with Coronavac produced the least IgG antibodies in both the cancer cohort and healthy cohort, across all timepoints. However, the difference in serum IgG titer between Coronavac and the other vaccines appeared to be diminished at TP4 in both graphs.

**Table 3:** Mean log IgG titer, in antibody units (U/ml), and 95% CI among the cancer cohort and healthy cohort by vaccine type ^1^TP1: pre-first dose, TP2: pre-second dose, TP3: 2 weeks post-second dose, TP4: 6 months post-first dose.

Time point^1^	Cancer patients, antibody units (95% confidence interval)			Healthy individuals, antibody units (95% confidence interval)	
	BNT162b2	AZ1222	Coronavac	BNT162b2	Coronavac
TP1	-0.18 (-0.39; 0.04)	2.31 (0.77; 3.86)	-0.18 (-2.08; 1.72)	-0.56 (-0.78; -0.34)	-0.80 (-0.96; -0.64)
TP2	2.78 (2.47; 3.09)	4.37 (3.33; 5.42)	-0.37 (-1.29; 0.54)	4.36 (4.11; 4.61)	1.07 (0.85; 1.29)
TP3	6.25 (5.98; 6.51)	7.19 (6.56; 7.82)	1.83 (0.27; 3.40)	7.33 (7.13; 7.52)	4.03 (3.74; 4.33)
TP4	7.07 (6.73; 7.40)	6.08 (5.37; 6.78)	5.87 (2.41; 9.34)	8.54 (8.14; 8.95)	6.38 (6.06; 6.70)

The results of linear mixed-model regression analysis within the cancer cohort are shown in Table 4. Patients who received AZ1222 were excluded from the analysis since it was not possible to disentangle the effect of pre-vaccine infection, given that almost all patients receiving AZ1222 had pre-vaccine infection. Patients with cancer who received the Coronavac vaccine produced fewer IgG antibodies than those who received the BNT162b2 vaccine (-3.09, 95% confidence interval=-4.37 to -1.80, p<0.01). Among the healthy cohort, patients who received the Coronavac vaccine also produced fewer IgG antibodies than patients who received the BNT162b2 vaccine (-2.64, 95% confidence interval=-2.97 to -2.30, p<0.01).

**Table 4:** Linear mixed-model regression of the effect of type of vaccine on log serum IgG levels ^1^Adjusted for pre-vaccine infection, age, ethnicity, and time point, random intercept for the individual. ^2^Adjusted for pre-vaccine infection, age, ethnicity, comorbidity, time point, income, cancer type, and booster, random intercept for the individual. ^3^Adjusted for pre-vaccine infection, age, ethnicity, comorbidity, time point, and booster, random intercept for the individual.

	Univariate, coefficient (95% confidence interval)		Model A^1^, coefficient (95% confidence interval)		Model B^2^, coefficient (95% confidence interval)	
All cancer patients	Estimate	P-Value	Estimate	P-Value	Estimate	P-Value
Type of vaccine						
BNT162b2	Ref		Ref		Ref	
Coronavac	-2.93 (-4.22; -1.65)	<0.01	-3.00 (-4.29; -1.71)	<0.01	-3.09 (-4.37; -1.80)	<0.01
	Univariate		Model A^1^		Model B^2^	
Only cancer patients diagnosed in 2020/2021	Estimate	P-Value	Estimate	P-Value	Estimate	P-Value
Type of vaccine						
BNT162b2	Ref		Ref		Ref	
Coronavac	-2.16 (-4.10; -0.22)	0.03	-2.23 (-3.94; -0.51)	0.01	-2.09 (-3.72; -0.45)	0.01
	Univariate		Model A^1^		Model C^3^	
Healthy individuals	Estimate	P-Value	Estimate	P-Value	Estimate	P-Value
Type of vaccine						
BNT162b2	Ref		Ref		Ref	
Coronavac	-2.82 (-3.06; -2.58)	<0.01	-2.63 (-2.90; -2.36)	<0.01	-2.64 (-2.97; -2.30)	<0.01

Since the cancer cohort also included long-term survivors, we performed a sensitivity analysis limited to cancer patients who were recently diagnosed in 2020 or 2021. When the linear mixed-model regression analysis was limited to this subgroup, patients who received the Coronavac vaccine still had lower IgG levels than patients who received the BNT162b2 vaccine (-2.09, confidence interval=-3.72 to -0.45, p=0.01).

**Figure 4:**
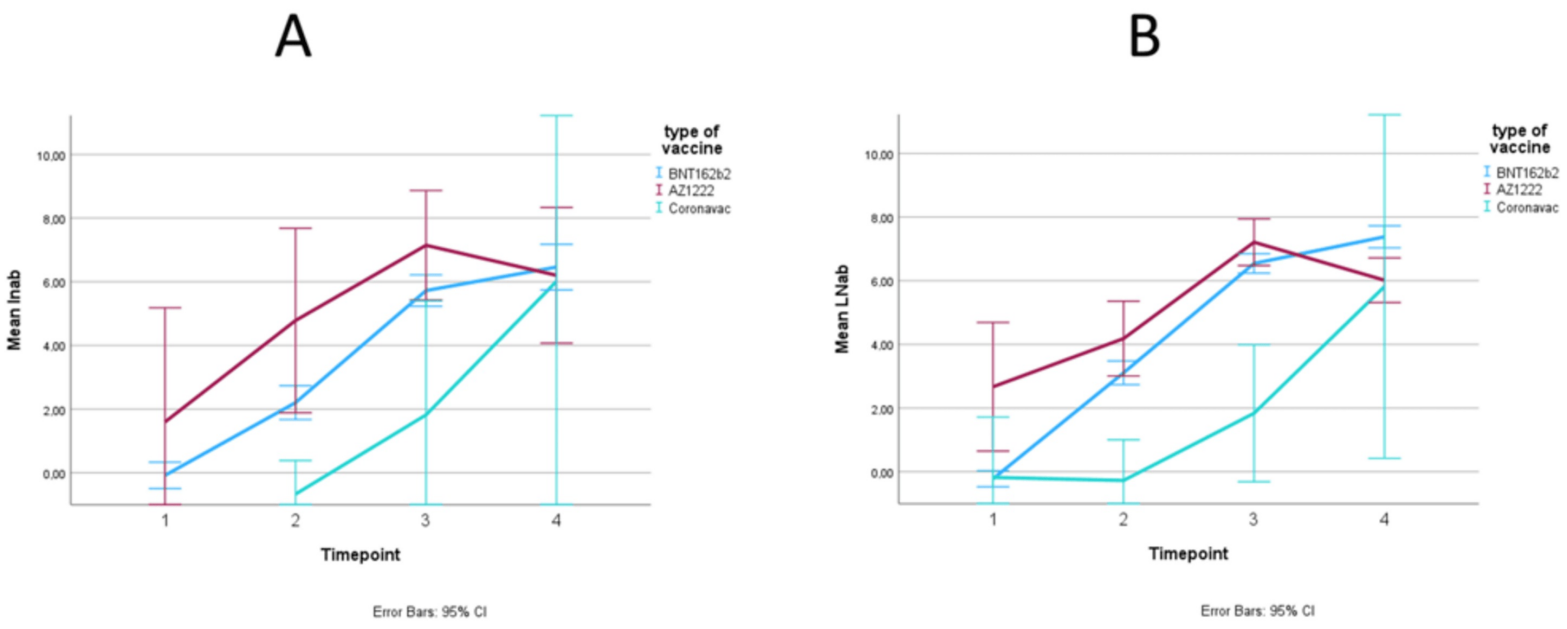
Trend in mean log IgG titer over time points, in antibody units, stratified by sex (A: men, B: women) in the cancer cohort

**Figure 5:**
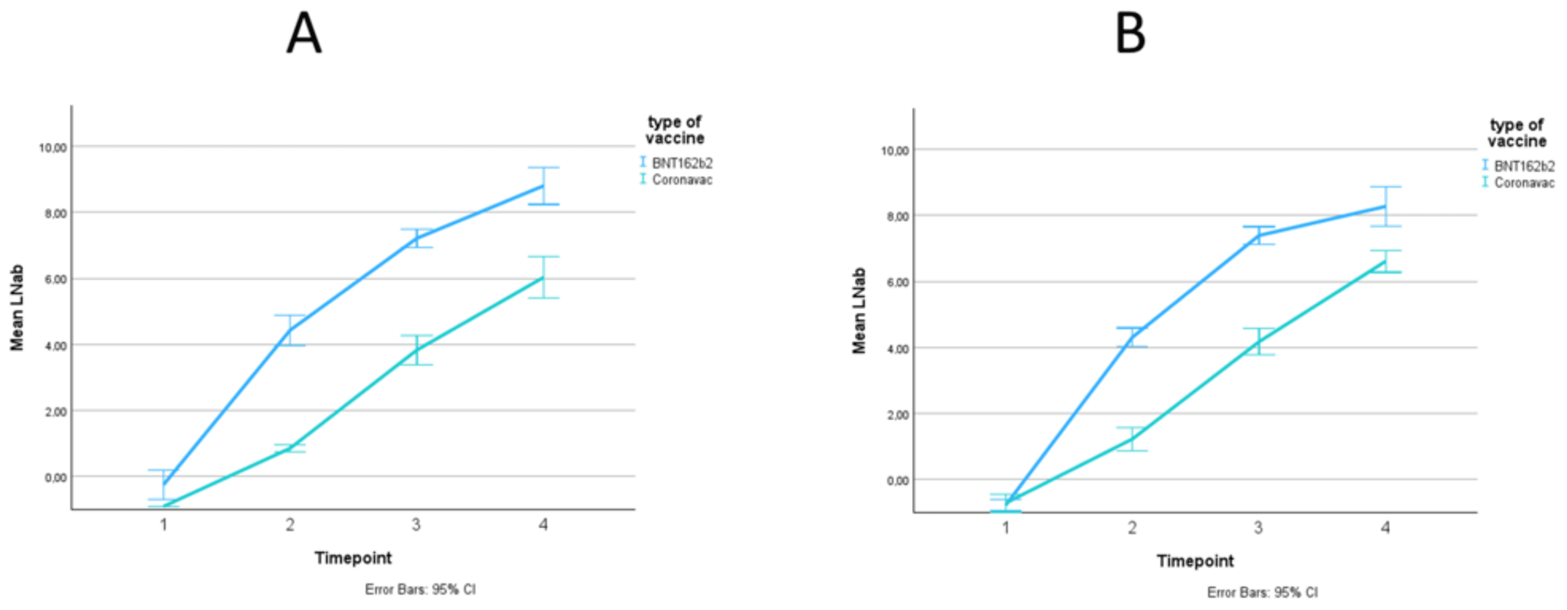
Trend in mean log IgG titer over time points, in antibody units, stratified by sex (A: men, B: women) in the healthy cohort

The difference in antibody response by vaccine type among cancer patients, stratified for sex, is illustrated in Figure 4. The difference in antibody response by vaccine type among healthy individuals, stratified for sex, is illustrated in Figure 5. The trends in serum IgG titer by vaccine type did not differ among males and females. The formal testing of sex as an effect modifier is shown in Table 5. Sex did not modify the antibody response to different vaccine types, both in the cancer cohort (Wald p=0.54) and in the healthy cohort (Wald p=0.94).

**Table 5:** Linear mixed-model regression of the effect of type of vaccine on log serum IgG level among cancer patients, investigating sex as an effect modifier ^1^Adjusted for pre-vaccine infection, age, ethnicity, time point, comorbidities, income, cancer type, and booster. ^2^Adjusted for pre-vaccine infection, age, ethnicity, time point, comorbidities, income, cancer type, booster, and an interaction term for vaccine and sex. ^3^Adjusted for pre-vaccine infection, age, ethnicity, comorbidities, booster, and time point. ^4^Adjusted for pre-vaccine infection, age, ethnicity, comorbidities, booster, time point, and an interaction term for vaccine and sex.

	Model without sex as an effect modifier^1^, coefficient (95% confidence interval)		Model with sex as an effect modifier^2^, coefficient (95% confidence interval)	
Cancer patients	Estimate	P-Value	Estimate	P-Value
Type of vaccine				
BNT162b2	Ref		Ref	
Coronavac	-3.13 (-4.41; -1.85)	<0.01	-2.45 (-4.87; -0.03)	0.05
Sex				
Male	Ref		Ref	
Female	0.39 (-0.11; 0.90)	0.12	0.42 (-0.11; 0.95)	0.12
Interaction				
Vaccine*Sex			-0.91 (-3.60; 1.78)	0.51
Wald test p-value				0.54
	Model without sex as an effect modifier^3^		Model with sex as an effect modifier^4^	
Healthy individuals	Estimate	P-Value	Estimate	P-Value
Type of vaccine				
BNT162b2	Ref		Ref	
Coronavac	-2.67 (-2.99; -2.35)	<0.01	-2.68 (-3.20; -2.17)	<0.01
Sex				
Male	Ref		Ref	
Female	0.20 (-0.05; 0.45)	0.11	0.19 (-0.20; 0.58)	0.31
Interaction				
Vaccine*Sex			0.02 (-0.52; 0.56)	0.93
Wald test p-value				0.94

The adverse events following vaccination are summarized in Table 6. In the cancer cohort, 35 patients (9.0% of the total) reported at least one adverse event following the first dose, with 34 of them receiving BNT162b2 and one receiving AZ1222. Following the second dose, 38 patients reported at least one adverse event (9.8% of the total), with all of them receiving BNT162b2. The most common adverse event was pain at the injection site. We did not have data on adverse events in the healthy cohort.

**Table 6:** Adverse events following vaccination in cancer patients

Adverse events	After the first dose	After the second dose
Total	35	39
Erythema	0	1
Pain at the injection site	20	13
Swelling at the injection site	5	2
Lymphadenopathy	0	0
Flu-like symptoms	2	0
Headache	5	12
Chills	3	3
Fatigue	15	11
Fever	3	8
Myalgia	2	1
Arthralgia	2	5
Nausea	1	2
Vomiting	1	2
Diarrhea	2	1
Other	3	10

Among the cancer cohort, 76 patients (19.5%) reported that they developed COVID-19 infections after vaccination. Four of the cancer patients were hospitalized for these infections. Among the healthy cohort, 33 (13.4%) participants reported that they developed COVID-19 infections after vaccination. The factors associated with breakthrough infections are shown in Table 7. Among the healthy individuals, Coronavac was associated with higher odds of breakthrough infection (odds ratio=7.34, confidence interval=1.40 to 33.49, p=0.02). Higher serum IgG at TP3 was associated with lower odds of breakthrough infections among patients with cancer (odds ratio=0.80, confidence interval=0.65 to 0.99, p=0.04).

**Table 7:** Logistic regression of the effects of variables on the odds ratio of breakthrough infection ^1^Adjusted for age, ethnicity, sex, vaccine type, and serum IgG after the second vaccine. ^2^Adjusted for age, ethnicity, sex, vaccine type, serum IgG after the second vaccine, comorbidity, income, cancer type, and booster. ^3^Adjusted for age, ethnicity, sex, vaccine type, serum IgG after the second vaccine, comorbidity, and booster.

	Univariate, odds ratio (95% confidence interval)		Model A^1^, odds ratio (95% confidence interval)		Model B^2^, odds ratio (95% confidence interval)	
Cancer patients	Estimate	P-Value	Estimate	P-Value	Estimate	P-Value
Type of vaccine						
BNT162b2	Ref		Ref		Ref	
Coronavac	NA		NA		NA	
Log serum IgG after the second vaccine						
Per unit increase	0.83 (0.73; 0.95)	0.01	0.80 (0.67; 0.94)	0.01	0.80 (0.65; 0.99)	0.04
	Univariate		Model A^1^		Model C^3^	
Healthy individuals	Estimate	P-Value	Estimate	P-Value	Estimate	P-Value
Type of vaccine						
BNT162b2	Ref					
Coronavac	1.62 (0.77; 3.45)	0.21	7.54 (1.71; 33.26)	0.01	7.34 (1.40; 33.49)	0.02
Log serum IgG after the second vaccine						
Per unit increase	1.02 (0.84; 1.23)	0.85	1.28 (0.88; 1.86)	0.19	1.30 (0.88; 1.92)	0.19

## Discussion

In this multiethnic, middle-income setting, it appeared that two COVID-19 vaccines (BNT162b2 and Coronavac) that were introduced under the National COVID-19 Immunisation Programme were associated with high seroconversion proportions and high IgG titers at six months. Nonetheless, vaccination with BNT162b2 yielded the highest IgG titer in cancer patients and healthy individuals, whereas Coronavac was associated with a lower antibody response. Antibody response was not modified by sex. We were unable to perform regression analysis with AZ1222 since we could not disentangle the effect of pre-vaccine infection, as almost all AZ1222 patients were exposed to COVID-19 beforehand.

Coronavac was associated with lower IgG titer than those who received BNT162b2 among patients with cancer. Our finding is in line with previous research on immunocompromised patients. A previous study examining antibody response following COVID-19 vaccination in patients with multiple sclerosis for instance observed that those who received Coronavac had lower antibody levels than their counterparts receiving BNT162b2 [19].

We also observed that healthy individuals who received Coronavac produced fewer antibodies than those who received BNT162b2. Previous research on the Malaysian general population observed that Coronavac had lower vaccine effectiveness against COVID-19 infection than BNT162b2 [20]. While our study and the aforementioned study had different outcomes (antibody titer and vaccine effectiveness, respectively), previous research has shown a correlation between antibody titer and vaccine effectiveness [21].

We observed that the effect of different vaccine types was not modified by sex. However, a previous observational study on 2.5 million Mexican pensioners suggested that vaccines, regardless of type, may be more effective in females than males [22]. A systematic review and meta-analysis of COVID-19 vaccine clinical trials, including adenoviral vector and mRNA vaccines, nonetheless found no significant effect modification by sex [23]. The contradicting evidence in this area suggests that further research, which is specifically designed to investigate the effect of sex on COVID-19 vaccine effectiveness, is warranted.

Coronavac vaccine was associated with higher odds of breakthrough infection than BNT162b2 in the healthy cohort, while we did not have enough data to make conclusions in the cancer cohort. Even so, the different measurements of breakthrough infections, by definition and timing, in the cancer and healthy cohorts mean that they are not directly comparable, as COVID-19 variants and breakthrough effects change over time [24,25]. Previous research suggests that BNT162b2 remains effective in preventing breakthrough infections (vaccine effectiveness 65.2%) among healthy Malaysians [26]. However, we could not find a similar study on patients with cancer, suggesting that this could be a focus for further research.

A strength of our study is the follow-up period of 26 weeks, which gives insight into the mid-term effects of vaccination on serum IgG titers. Data were collected prospectively, minimizing the risk of bias associated with retrospective cohort studies [27]. Additionally, we directly measured antibody quantities in the blood, offering greater insights than using a proxy, or binary seroconversion outcome (yes, no). However, a limitation of our study is the high attrition rate at one year of follow-up, which prevented analysis at that time point. The relatively small sample size of patients with cancer patients who received Coronavac also limits the statistical power of our analysis. As this is an observational study rather than a randomized controlled trial, we can only make statements on correlation or association, not causality. Regardless, we are confident that our results are generalizable, particularly to cancer patients based on cancer type or stage. Future research could focus on head-to-head comparisons between different vaccines or treatment strategies, with a larger sample of patients receiving Coronavac in order to provide deeper insights.

Regardless, the present study adds to the body of real-world evidence on antibody response to COVID-19 vaccines in diverse populations. From the perspective of a multiethnic, LMIC setting, it appears that all three vaccines that were introduced under the National COVID-19 Immunisation Programme in Malaysia were able to elicit a sufficient antibody response in the population including in individuals with cancer. Our study further provides evidence that the antibody response of different types of vaccines is not modified by sex.

## Conclusions

In this multiethnic, middle-income setting, BNT162b2 and Coronavac vaccines appear to induce high antibody titers and seroconversion rates including in individuals with cancer. Sex did not modify antibody response. Findings further suggest that vaccination with BNT162b2 yields the highest IgG titer compared to other vaccines, in both healthy individuals and those with cancer.

## References

[1] Li M, Wang H, Tian L (2022). COVID-19 vaccine development: Milestones, lessons and prospects. Signal Transduct Target Ther.

[2] Dadras O, SeyedAlinaghi S, Karimi A (2022). COVID-19 vaccines' protection over time and the need for booster doses; a systematic review. Arch Acad Emerg Med.

[3] Stroppa EM, Toscani I, Citterio C, Anselmi E, Zaffignani E, Codeluppi M, Cavanna L (2020). Coronavirus disease-2019 in cancer patients. A report of the first 25 cancer patients in a western country (Italy). Future Oncol.

[4] Corti C, Antonarelli G, Scotté F (2022). Seroconversion rate after vaccination against COVID-19 in patients with cancer-A systematic review. Ann Oncol.

[5] Lee AR, Wong SY, Chai LY (2022). Efficacy of COVID-19 vaccines in immunocompromised patients: Systematic review and meta-analysis. BMJ.

[6] Yusuf A, Sarfati D, Booth CM (2021). Cancer and COVID-19 vaccines: A complex global picture. Lancet Oncol.

[7] Xu K, Wang Z, Qin M (2023). A systematic review and meta-analysis of the effectiveness and safety of COVID-19 vaccination in older adults. Front Immunol.

[8] Antwi J, Appiah B, Oluwakuse B, Abu BA (2021). The nutrition-COVID-19 interplay: A review. Curr Nutr Rep.

[9] Heidari S, Babor TF, De Castro P, Tort S, Curno M (2016). Sex and gender equity in research: Rationale for the SAGER guidelines and recommended use. Res Integr Peer Rev.

[10] Azami NA, Perera D, Thayan R (2022). SARS-CoV-2 genomic surveillance in Malaysia: Displacement of B.1.617.2 with AY lineages as the dominant Delta variants and the introduction of Omicron during the fourth epidemic wave. Int J Infect Dis.

[11] (2024). T20, M40, B40 Household Income Update. https://www.mef.org.my/Attachments/MSN20230731a.pdf.

[12] Riester E, Findeisen P, Hegel JK (2021). Performance evaluation of the Roche Elecsys Anti-SARS-CoV-2 S immunoassay. J Virol Methods.

[13] (2024). Elecsys® Anti-SARS-CoV-2 S Assay Method Sheet. https://www.fda.gov/media/144037/download/.

[14] Fu JY, Pukhari MH, Bador MK, Sam IC, Chan YF (2023). Humoral and T cell immune responses against SARS-CoV-2 after primary and homologous or heterologous booster vaccinations and breakthrough infection: A longitudinal cohort study in Malaysia. Viruses.

[15] IBM Corp (2023). IBM SPSS Statistics for Windows, Version 29.0.

[16] RStudio Team. RStudio (2015). Integrated Development Environment for R [Internet]. Boston, MA. http://www.rstudio.com/.

[17] Rubin DB, Schenker N (1991). Multiple imputation in health-care databases: An overview and some applications. Stat Med.

[18] Eekhout I, van de Wiel MA, Heymans MW (2017). Methods for significance testing of categorical covariates in logistic regression models after multiple imputation: Power and applicability analysis. BMC Med Res Methodol.

[19] Tütüncü M, Demir S, Arslan G (2023). mRNA versus inactivated virus COVID-19 vaccines in multiple sclerosis: Humoral responses and protectivity-Does it matter?. Mult Scler Relat Disord.

[20] Suah JL, Husin M, Tok PS (2022). Waning COVID-19 vaccine effectiveness for BNT162b2 and CoronaVac in Malaysia: An observational study. Int J Infect Dis.

[21] Liu J, Bodnar BH, Padhiar NH (2021). Correlation of vaccine-elicited antibody levels and neutralizing activities against SARS-CoV-2 and its variants. Clin Transl Med.

[22] Hernandez-Avila M, Ortiz-Brizuela E, Tamayo-Ortiz M (2023). Assessing the real-world effectiveness of five SARS-CoV-2 vaccines in a cohort of Mexican pensioners: A nationwide nested test-negative design study. Lancet Reg Health Am.

[23] Zhu Z, Xu L, Chen G (2021). Is there a difference in the efficacy of COVID-19 vaccine in males and females? - A systematic review and meta-analysis. Hum Vaccin Immunother.

[24] Sam IC, Chong YM, Abdullah A (2022). Changing predominant SARS-CoV-2 lineages drives successive COVID-19 waves in Malaysia, February 2020 to March 2021. J Med Virol.

[25] Doke PP, Mhaske ST, Oka G, Kulkarni R, Muley V, Mishra AC, Arankalle VA (2022). SARS-CoV-2 breakthrough infections during the second wave of COVID-19 at Pune, India. Front Public Health.

[26] Lim AH, Ab Rahman N, Ong SM (2022). Evaluation of BNT162b2 vaccine effectiveness in Malaysia: Test negative case-control study. Vaccine.

[27] Andrade C (2022). Research design: Cohort studies. Indian J Psychol Med.

